# The Replacement of the Teeth

**Published:** 1853-07

**Authors:** C. F. Cushman


### ARTICLE VI.

### *The Replacement of the Teeth.* By C. F. CUSHMAN, D. D.

NO. II.

IN the January No. of the *Journal*, I gave some illustrations of the practice of *replacement of luxated teeth*—a subject which, I doubted not, would be regarded as *heretical* by many; by some as too *antiquated* a practice for this modern and progressive age, if nothing worse. All such, and all those who have witnessed only unfavorable cases, or none at all, I presume were fully disposed to reject it entirely.

But, believing that the true mission of "the healing art" is to *assist*, rather than supplant nature, I am willing to accept truth wherever I find it; and am, therefore, practically "eclectic."

I have now to add another case, eminently showing the practical advantage of replacing a luxated tooth.

CASE 3. Miss M., aet. about twenty-five, temperament sanguino-lymphatic, health robust, denture regular and symmetrical; applied to a dentist four years previous to her visiting me, and requested him to extract the second bicuspis, right side, of the inferior jaw, it being diseased and painful.

He made six trials at it—"I tell the tale as 'twas told me"—and on the *seventh*, (there wasn't "luck in odd numbers" here!) by some strange fatality, or unaccountable carelessness—for it was daytime, he seized hold of the *first bicuspis*, which was perfectly sound, and extracted *it!*

The ejectment was complete, for she had the tooth in her hand during some two minutes; when learning the *mistake*, she ordered it *replaced*, and declined having the other out.*

She closed her jaws firmly upon it and drove it *home*, her superior denture being perfect. With no other means of re-

* It was extracted afterwards.

taining it in place, she repeated this movement, frequently, for several days. During the first three or four subsequently, it was very sore; for a whole week it gave *some* annoyance; but after that period very little, if any.

Up to the present time, now four years gone, it has never given rise to any abscess, or discharge of the gum. It is now quite as firm as any tooth—the gum looks pale and perfectly healthy—the color of the tooth (her teeth are very beautiful) is as *lively* as its neighbors—indeed, on close scrutiny, it is difficult to believe that it has ever been dislocated, since occular evidence thereof is wanting, if we except a slight opaque spot on the labial surface of the enamel, at the neck. That spot is the mark of the extracting instrument, the pressure of which disintegrated the chrystalline structure, and is itself a corroboration of the patient's statement of these remarkable facts.

Now, with such evidence of the practicability and success of replacing luxated teeth, I feel it to be the duty of every practitioner to make all reasonable efforts to *save* such as may come under his care—notwithstanding this peculiar practice may be denounced as *unscientific*, and the opinion be concurred in by patients themselves, too few of whom justly appreciate their natural endowments.

DON QUIXOTE did not exaggerate when he said "a diamond is not so precious as a tooth," and it was those of *nature's* gift whose loss he was bewailing. As to practice, that which is natural is scientific.

I have never seen, nor expect to see, the product of art, which as a dental substitute could approach beyond a remote degree towards the perfection of utility, comfort and beauty which this *replaced* tooth exhibits; and doubtless will continue to do for many years in the future.

*June*, 1853.

